# ADO/hypotaurine: a novel metabolic pathway contributing to glioblastoma development

**DOI:** 10.1038/s41420-020-00398-5

**Published:** 2021-01-22

**Authors:** Dachuan Shen, Lili Tian, Fangyu Yang, Jun Li, Xiaodong Li, Yiqun Yao, Eric W.-F. Lam, Peng Gao, Bilian Jin, Ruoyu Wang

**Affiliations:** 1 Department of Oncology, Affiliated Zhongshan Hospital of Dalian University, 116001 Dalian, Liaoning P.R. China; 2 Department of Oncology, First Affiliated Hospital of Dalian Medical University, 116011 Dalian, Liaoning P.R. China; 3 Department of Neurosurgery, General Hospital of Northern Theater Command, 110015 Shenyang, Liaoning P.R. China; 4 Department of Neurosurgery, First Affiliated Hospital of Dalian Medical University, 116011 Dalian, Liaoning P.R. China; 5 Institute of Cancer Stem Cell, Cancer Center, Dalian Medical University, 116044 Dalian, Liaoning P.R. China; 6 Department of Thyroid and Breast Surgery, Affiliated Zhongshan Hospital of Dalian University, 116001 Dalian, Liaoning P.R. China; 7 Department of Surgery and Cancer, Imperial College London, London, W12 0NN UK; 8 Clinical Laboratory, Dalian Sixth People’s Hospital, 116031 Dalian, Liaoning P.R. China

**Keywords:** Cancer stem cells, Preclinical research

## Abstract

Significant advance has been made towards understanding glioblastoma metabolism through global metabolomic profiling. However, hitherto little is known about the role by which altered metabolism plays in driving the aggressive glioma phenotype. We have previously identified hypotaurine as one of the top-ranked metabolites for differentiating low- and high-grade tumors, and that there is also a strong association between the levels of intratumoral hypotaurine and expression of its biosynthetic enzyme, cysteamine (2-aminoethanethiol) dioxygenase (ADO). Using transcription profiling, we further uncovered that the ADO/hypotaurine axis targets CCL20 secretion through activating the NF-κB pathway to drive the self-renewal and maintenance of glioma ‘cancer stem cells’ or glioma cancer stem-like cells. Conversely, abrogating the ADO/hypotaurine axis using CRISPR/Cas9-mediated gene editing limited glioblastoma cell proliferation and self-renewal in vitro and tumor growth in vivo in an orthotopical mouse model, indicating that this metabolic pathway is a potential key therapeutic target. Collectively, our results unveil a targetable metabolic pathway, which contributes to the growth and progression of aggressive high-grade gliomas, as well as a novel predictive marker for glioblastoma diagnosis and therapy.

## Introduction

Gliomas are the most common malignant brain tumors in adults, which arise primarily from glial tissue. The World Health Organization classifies gliomas into grades ranging from I to IV based on specific pathologic criteria. Current treatment options commonly involve multimodal therapy regimens, including surgery, ionizing radiation, and chemotherapy; however, these cancer therapeutic regimes are mostly palliative and not curative. Thus, the prognosis of glioma patients remains poor and only <3% of patients survive more than 5 years^1–3^. In consequence, novel insights into the molecular mechanisms for glioma tumorigenesis and the drivers of disease progression are urgently needed to expand our basic understanding and treatment options for gliomas.

Studies in cancer cell metabolism have extended our comprehension of the mechanisms of functional dysregulation during tumorigenesis and cancer progression. Metabolic reprogramming is a central hallmark of most cancers, which also include brain tumors. As a result, metabolite profiling has become a promising approach for uncovering novel clinical diagnostics and prognostics, as well as treatment strategies^4,5^. Therein, cysteine metabolism is known for its role in the synthesis of glutathione (GSH), which protects glioma against redox stress and hypoxia^6^. Cysteine itself is a semi-essential amino acid that subsequently catabolizes to glutathione and taurine, which also function as substantial intermediates for modulating brain function^7,8^. Taurine, is one of the most abundant amino acids in the human body and has a physiological role as an osmotic pressure controller^9–12^, a neuromodulator^13,14^, an immunomodulatory factor^15^, and an antioxidant for detoxifying hypochlorous acid to form taurine chloramine^13^. Taurine deficiency is associated with brain development abnormalities^13,16,17^. Hypotaurine is a sulfinic acid and an intermediate in the biosynthesis of taurine. The physiological roles of taurine and its precursor, hypotaurine, in glioma progression remain enigmatic.

In mammals, hypotaurine can be synthesized by two distinct pathways: (1) the conversion of cysteine to cysteine sulfinate by cysteine dioxygenase (CDO1), followed by its decarboxylation to hypotaurine by cysteine sulfinic acid decarboxylase (CSAD), and (2) the incorporation of cysteine into CoA, followed by the release of cysteamine during CoA turnover, and the oxidation of cysteamine to hypotaurine by ADO^18,19^. Through metabolomic profiling using a high-throughput capillary electrophoresis-mass spectrometry (CE-MS) of a total of 50 patient-derived glioma specimens from our previous study, we identified a pathway involved in cysteine catabolism and hypotaurine accumulation. We further demonstrated that hypotaurine to be one of the top-ranked metabolites for differentiating glioblastomas (GBMs) from low-grade gliomas and that there was also a strong association between the intratumoral hypotaurine levels and the expression levels of its biosynthetic enzyme cysteamine (2-aminoethanethiol) dioxygenase (ADO)^17^. In agreement, another previous metabolomic profiling study using gas chromatography-based mass spectrometry on a total of 69 patient-derived glioma specimens has also identified cysteine sulfinic acid (CSA) to be one of the top metabolites for differentiating low- and high-grade gliomas, with a >23-fold increase in CSA levels in gliobalstomas compared with grade 2 gliomas and a 6.63-fold increase in hypotaurine levels in glioblastomas^20,21^. In addition, the study also uncovered that there was also a strong association between the intratumoral hypotaurine levels and the expression levels of one of its upstream biosynthetic enzymes, cysteine dioxygenase (CDO1) in glomas, suggesting the CDO1/CSA axis might have a role in glioma progression^20,21^. In agreement, recent evidence has also suggested a role for hypotaurine in cancer tumorigenesis^22–24^. More recently, ADO has also been demonstrated to promote the oxidation of N-terminal cysteinyl residues to sulfinic acid and thereby proteasomal degradation in plant and animal cells^25^. Hitherto, the role of ADO, the enzyme responsible for the t biosynthesis of hypotaurine in a parallel pathway to CDO1/CSA, in glioma tumorigenesis and progression remains undefined.

Here, we report the discovery of the ADO/hypotaurine axis, a pathway involved in cysteine catabolism, augments the release of chemokine CCL20 via activating the NF-κB pathway. In addition, the accumulating CCL20 also further stimulates the NF-κB pathway to form a feedforward loop to promote glioma stem cell (GSC) renewal and maintenance. Collectively, our findings uncover that the ADO/hypotaurine axis promotes the stemness of glioma in NF-κB-CCL20 feedforward loop to drive GBM progression.

## Results

### ADO acts as an oncogene in glioma and its expression associates with tumor malignancy

**Fig. 1:**
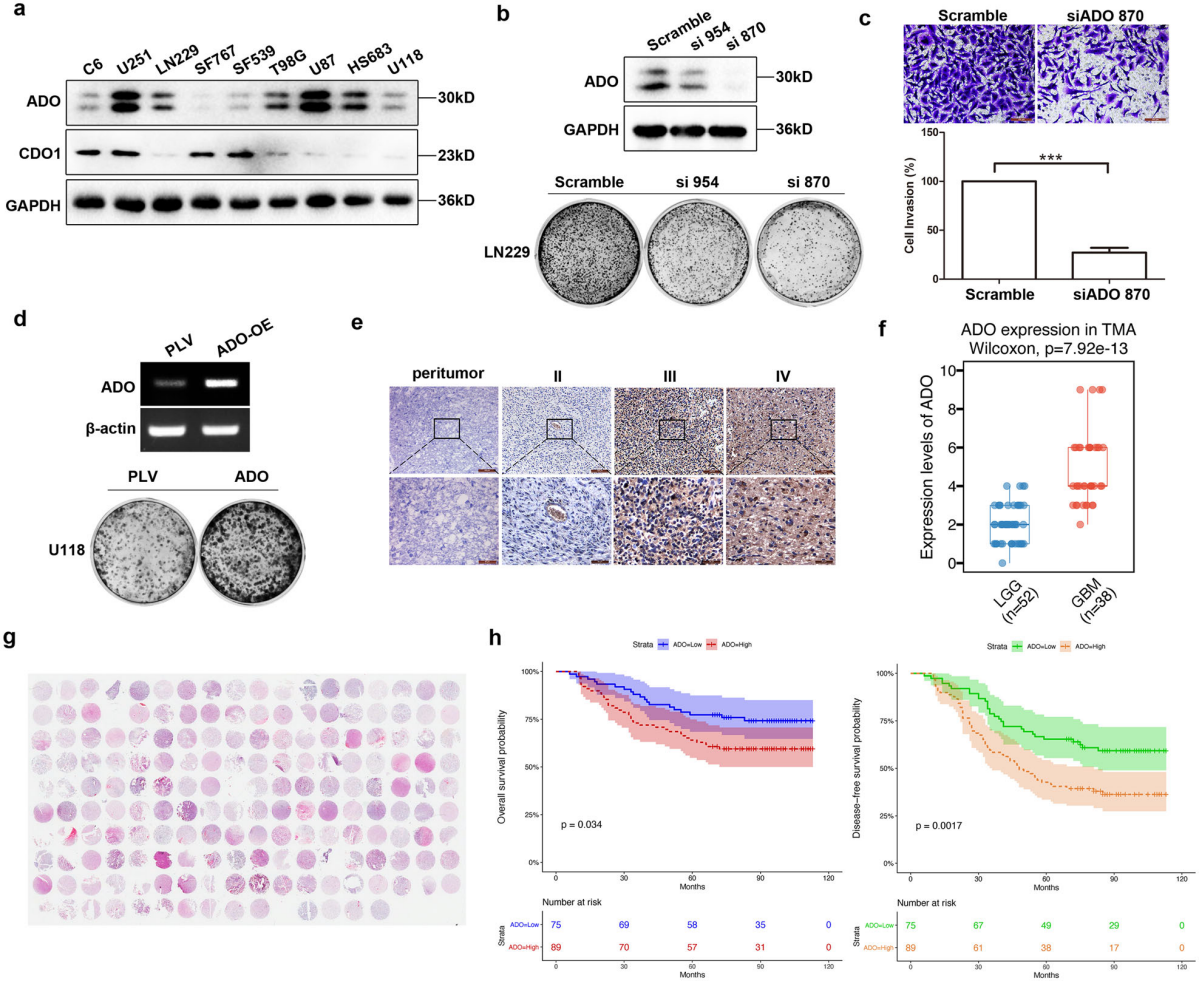
ADO functions as an oncogene in glioma. **a** ADO and CDO1 protein levels in 9 glioma cell lines. **b**, **c** siRNA of ADO (870, 954) corresponding to a decrease in clonal growth and invasion of LN229 cells. **d** overexpression (OE) ADO in U118 and SF767 cells corresponding to clonal growth. **e** ADO expression levels in 108 glioma patient samples and 18 normal brain sections obtained from intracranial aneurysms and peritumor. Immunoreactive score (IRS) based on staining intensity and distribution was then used to evaluate ADO expression in low-grade gliomas (LGG) and GBMs (**f**). Scale bars = 100 μm and 50 μm. **g** ADO is highly expressed in GBM in a cohort consisting of 180 glioma tissues, localized to the cytoplasm, and negatively correlated with the overall survival rate and disease-free survival of glioma patients (**h**). All results are representative of three independent experiments.

We have previously shown that intratumoral hypotaurine levels positively correlate with glioma grade and that there is also a strong association between the levels of intratumoral hypotaurine and expression of its biosynthetic enzyme ADO in glioma^17^. To explore the relevance of ADO expression in glioma progression, we first evaluated the expression of ADO and CDO1, enzymes involved in hypotaurine synthesis, in a panel of glioblastoma cell lines. ADO was differentially expressed in nine cell lines, with relatively high levels in U87 and LN229 cells, and low levels in U118 and SF767 cells (Fig. 1a). By comparison, CDO1 was only abundantly expressed in four out of the nine cell lines. The results suggested that ADO, like CDO1, may play an essential role in glioma progression. This led us to examine the role of ADO in glioma tumorigenesis and progression by investigating the effects of ADO depletion and overexpression in the glioma cell lines with high and low ADO expression, respectively. Knockdown of ADO expression by siRNA significantly inhibited LN229 cell growth and invasiveness (Fig. 1b, c), while overexpression of ADO substantially accelerated U118 cell proliferation (Fig. 1d). We next investigated further the hypothesis that ADO is required for GBM progression. To this end, we first studied by immunohistochemical staining ADO expression levels in a tissue microarray containing 108 glioma patient samples ranging from grade 2 to grade 4, and 18 normal control brain sections obtained from intracranial aneurysms and peritumor. The result showed that ADO expression was at significantly higher levels in grade 4 compared with grade 2 and 3 gliomas (Wilcoxon signed-rank test, *p* = 7.92 × 10^−13^), while little or no expression was detected in normal brain sections (Fig. 1e, f). Thereafter, the effects of ADO expression on patient survival was examined in a microarray with 180 glioma patient tissue samples and clinical data. Survival analysis indicated that high levels of ADO expression were strongly correlated with worse overall (*p = 0.034) and disease-free (**p = 0.0017) patient survival (Fig. 1g, h).

### ADO promotes the endogenous synthesis of hypotaurine

**Fig. 2:**
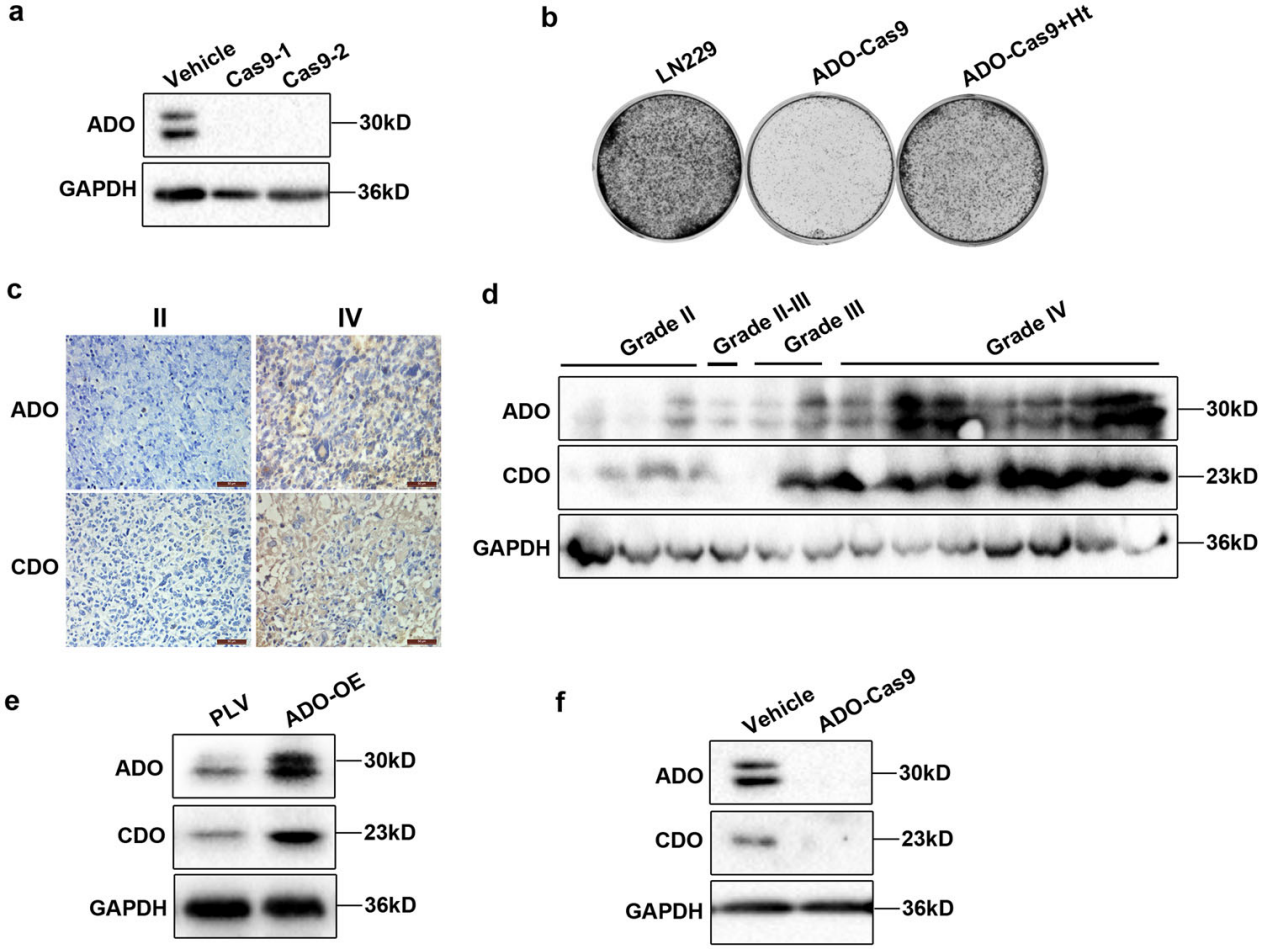
ADO drives the endogenous synthesis of hypotaurine in glioma. **a** ADO knockout cells were generated by utilizing CRISPR-Cas9 system in LN229. **b** Clone formation assays were conducted to evaluate the effect of ADO-Cas9 and hypotaurine on LN229 growth. **c**, **d** Serial sections or tumor tissue lysates of one patient were used to detect ADO and CDO expression by immunohistochemistry (IHC) and western blot, respectively. Scale bars = 50 μm. **e** Immunoblotting analysis of the CDO protein levels in U118 ADO-OE cells and **f** LN229 ADO-Cas9 cells. All results are representative of three independent experiments.

To better define the metabolic pathways and functional consequences associated with ADO/hypotaurine accumulation, we next generated ADO knockout cells utilizing the clustered regularly interspaced short palindromic repeats (CRISPR)-associated protein 9 (CRISPR-Cas9) system (Fig. 2a). Consistent with earlier results, ADO-Cas9 deletion remarkably attenuated the clonogenic ability of LN229 cells, which could be effectively rescued by co-treatment of hypotaurine (2 mM) (Fig. 2b). Endogenous hypotaurine derives from two synthetic pathways in which ADO and CDO1 serve as key enzymes; however, but their respective roles in gliomas are unclear. Of interest, we found that patients with high levels of ADO have parallel high expression of CDO1, while low expression of ADO along with low levels of CDO1 in patient tissue samples (Fig. 2c, d). Intriguingly, this strong and significant correlation between ADO and CDO1 was observed only at the protein level (Supplementary Fig. S1) but not at the mRNA transcript level^26^ (Supplementary Fig. S2), indicating the connection between ADO and CDO1 expression is mediated at the post-transcriptional level. Consistently, CDO1 was upregulated when ADO overexpressed in U118 cells (Fig. 2e), while CDO1 was coordinately downregulated to undetectable levels upon ADO deletion in LN229 cells (Fig. 2f). Collectively, these findings suggest that ADO regulates CDO1 expression in glioma.

### ADO is upregulated in glioblastoma stem-like cells

**Fig. 3:**
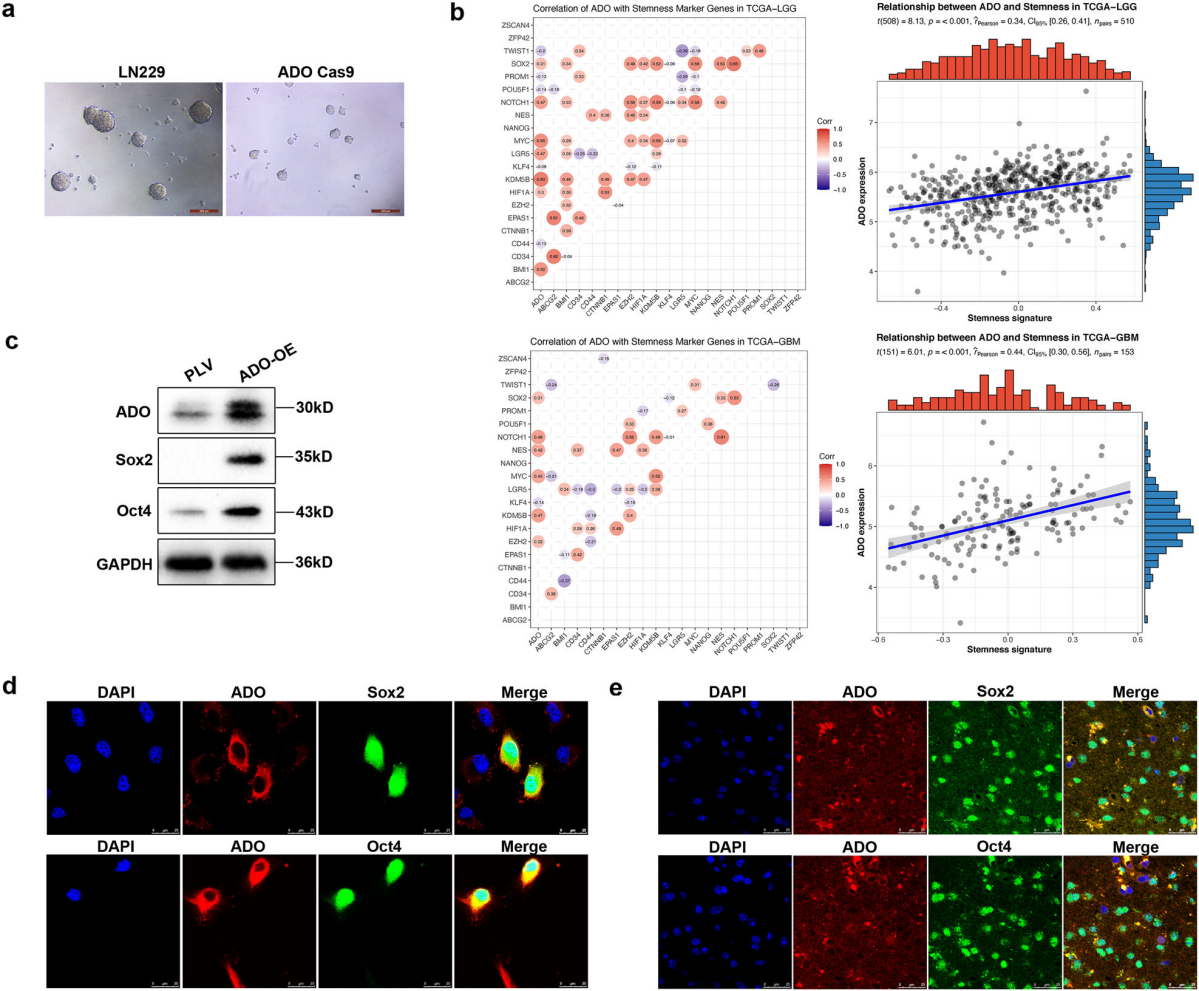
ADO is upregulated in glioblastoma stem-like cells. **a** Sphere formation derived from LN229 ADO-Cas9 and control cells. Scale bars = 200 μm. **b** Analysis of correlations between ADO and a series of stemness markers in two TCGA cohorts (TCGA-LGG and TCGA-GBM), which were retrieved from GDC data portal (https://portal.gdc.cancer.gov/). **c**, **d** Sox2 and Oct4 expression was detected by western blotting and immunofluorescence under confocal fluorescence microscopy in U118 and ADO-OE U118 cells, respectively. Scale bars = 25 μm. **e** Sox2, Oct4, and ADO co-staining in glioma patients sections with high levels of ADO, Sox2, and Oct4 were located within the nucleus. Scale bars = 25 μm. All results are representative of three independent experiments.

When ADO was overexpressed in U118 cells, we detected morphological changes in U118 from long spindly to more rounded cells (Supplementary Fig. S3). Moreover, knockout of ADO in LN229 cells notably reduced spheroid formation (Fig. 3a), implying that ADO is related to glioma clonogenicity and stem-like cell maintenance. Next, we studied the relationship between ADO and a series of established GSC markers from the TCGA database and found that ADO exhibited strong correlations with stemness signatures both in low-grade glioma (LGG) and GBM (Fig. 3b). Further examination revealed that U118 cells overexpressing ADO displayed higher levels of stemness markers (e.g., Sox2 and Oct4) compared to control U118 cells (Fig. 3c, d). Consistent with our cell culture findings, patients with high levels of ADO also expressed higher levels of Sox2 and Oct4 (Fig. 3e), which could not be detected in patients with low ADO expression (Supplementary Fig. S4). Collectively, overexpression of ADO promoted, while ADO elimination attenuated a set of the glioma stem cell phenotypes.

### ADO promotes glioma stemness via augmenting a NF-κB-CCL20 axis

**Fig. 4:**
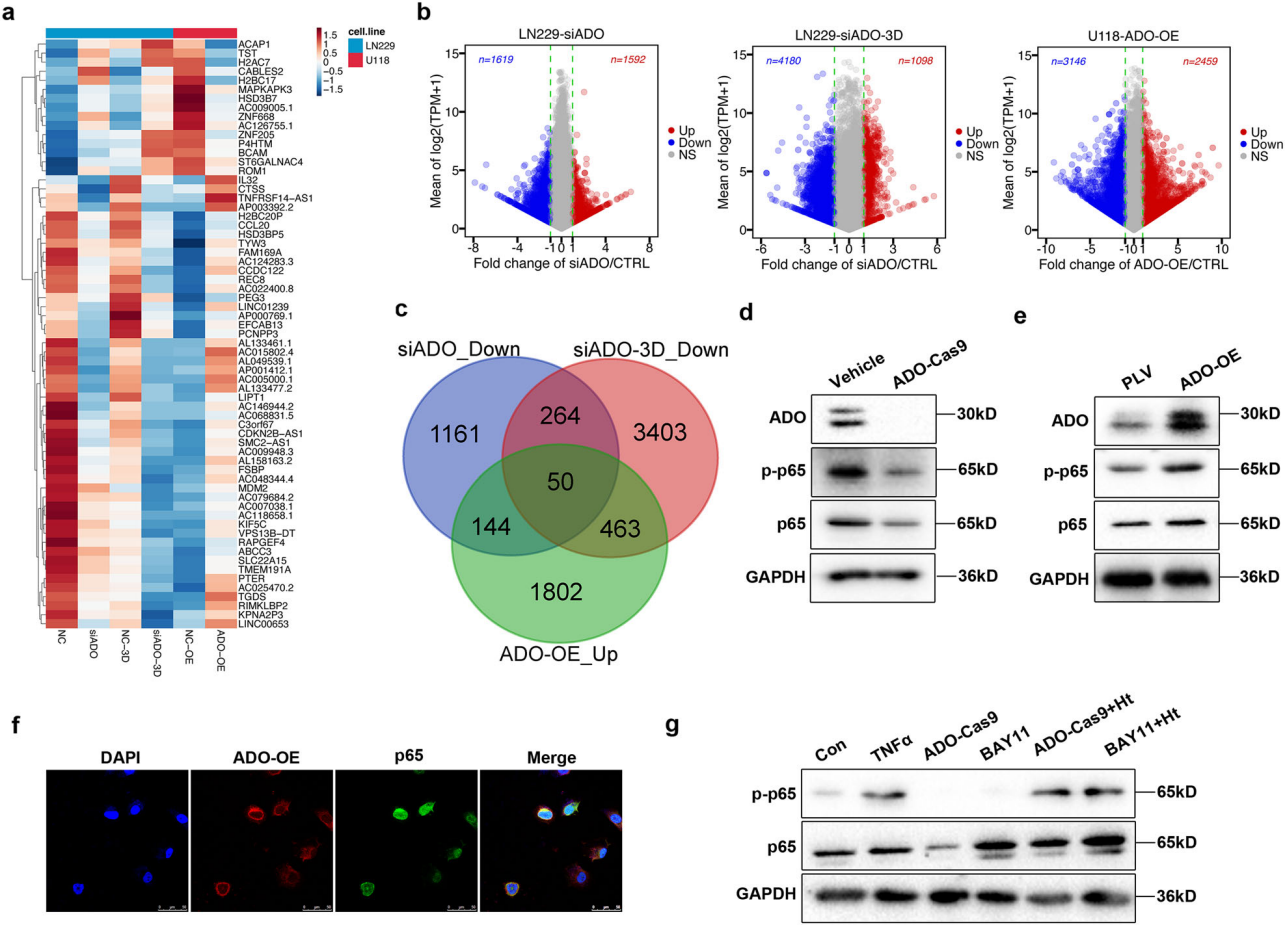
ADO promotes NF-κB pathway activation. **a**, **b** RNA-seq analysis in three paired experiments: ADO knockdown and controls under both 2D and 3D (LN229-siADO-derived spheroids) cultural conditions, and U118 ADO-OE cells and control groups. **c** Venn diagram representing overlapping candidates between three pairs of RNA-seq. **d** Phosphorylated p65 (p-p65) and total p65 proteins levels were analyzed in LN229 ADO-Cas9 cells and **e** U118 ADO-OE cells and their respective controls by immunoblotting. **f** ADO and p65 co-staining in U118 ADO-OE cells. Scale bars = 50 μm. **g** LN229 cells were treated with TNFα (2 μM) or NF-κB inhibitor BAY11-7082 (2 μM) to examine the effects of co-treatment of hypotaurine with ADO-Cas9 or BAY11-7082 on p-p65 and total p65 protein levels. Data represent three independent experiments with similar results.

To gain insights into the mechanisms by which ADO regulates the glioma stem cell phenotype, we first sought to identify the potential ADO downstream pathways—which have a role in glioma stem cell maintenance. To this end, we conducted RNA-seq enrichment analysis in three paired ADO knockdown and overexpression experiments: knockdown ADO with controls under both 2D and 3D (LN229-siADO-derived spheroids) cultural conditions, and overexpressing (OE) ADO in U118 cells with control cells (Fig. 4a, b). The heatmap revealed a significant enrichment for gene sets involved in the NF-κB pathway, a superfamily of small chemotactic cytokines (CCL20, CXCL1, CXCL2, CXCL5, CXCL16) and stem cell pluripotency (Fig. 4c). The RNA-seq data were validated independently using quantitative RT-PCR analysis of the mRNA levels of the five candidate genes most affected by ADO expression in the ADO-Cas9 versus control LN229 cells (Supplementary Fig. S5). In addition, we observed a strong correlation between the expression levels of ADO and NF-κB pathway members in the TCGA-GBM database (Supplementary Fig. S6). We thus hypothesized that ADO could promote GSCs through activating the NF-κB pathway. To test this conjecture, we examined the phosphorylated p65 (p-p65) protein levels, which reflect NF-κB activity in LN229 ADO-Cas9 and control cells. We found depletion of ADO expression contributed not only to attenuated p-p65 expression but also to decreased levels of total p65 (Fig. 4d). In agreement, ADO-OE contributed to substantial augments of both p-p65 and total p65 protein levels (Fig. 4e). Furthermore, immunofluorescence assay showed an evident p65 translocation into the nucleus due to ADO-OE in U118 cells (Fig. 4f), further suggesting that ADO functions as an upstream regulator of the NF-κB signaling pathway. Significantly, the observed inactivation of NF-κB as a result of ADO knockout or treatment with BAY11-7082 (2 μM), a NF-κB inhibitor, could be rescued by co-treatment with hypotaurine (2 mM) (Fig. 4g), further supporting the role this metabolite and its biosynthetic enzyme ADO in activating the NF-κB pathway.

**Fig. 5:**
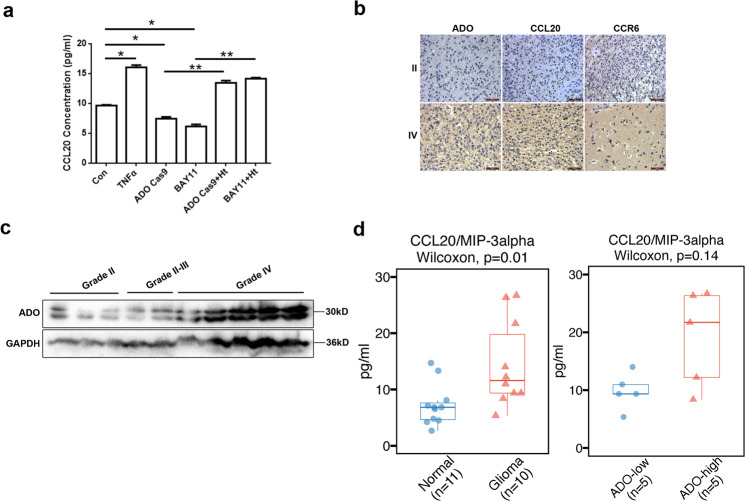
ADO promotes glioma stemness via augmenting a NF-κB-CCL20 feedforward activation loop. **a** The concentrations of CCL20 generated from 6 groups were detected using Elisa: LN229 control group, cells treated with TNFα (2 μM), ADO-Cas9 cells, cells paired with NF-κB inhibitor BAY11-7082 (2 μM), ADO-Cas9 cells companied by hypotaurine (2 mM), BAY11-7082 companied by hypotaurine (2 mM). **b** Serial sections from patients were used to study ADO, CCL20, and CCR6 expression by immunohistochemistry. Scale bars = 50 μm. Representative staining patterns are shown. **c** Western blot analysis of ADO expression in 10 glioma patients. Cerebrospinal fluid was collected from these 10 glioma patients with varying ADO levels to test for intracranial CCL20, CXCL1, CXCL2, CXCL5, and CXCL16 levels using Luminex assays (R&D Systems). **d** The concentrations of CCL20 were compared between in glioma patients and nontumor patients, and also in patients with high and low levels of ADO. Data represent three independent experiments with similar results. Student’s *t*-test, *p < 0.05, **p < 0.01.

Notably, in the aforementioned RNA-seq enrichment analysis, a superfamily of small chemotactic cytokines were downregulated with ADO siRNA-depletion under both 2D and 3D cultural conditions of LN229 cells and also upregulated with ADO overexpression in U118 cells. Among these chemotactic cytokines, chemokine C-C motif ligand 20 (CCL20) was the most prominently affected by ADO expression. We next validated and quantified the CCL20 levels in glioblastomas using Elisa. The result demonstrated 1–1.6- fold increase in CCL20 levels, which corresponded to CCL20 concentrations ranging from 9.6 to 16.4 pg/ml in cells with an active ADO-hypotaurine axis (Fig. 5a). Specifically, we observed an increase in CCL20 concentrations with the administration of TNFα (2 μM), which induces the NF-κB pathway. Conversely, LN229 ADO-Cas9 depletion and treatment with the NF-κB pathway inhibitor BAY11-7082, caused a significant reduction in CCL20 levels. However, treatment with hypotaurine (2 mM) effectively restored the endogenous CCL20 levels (Fig. 5a). These results demonstrated that the secretion of CCL20 can be prevented by downregulation of ADO or inactivation of the NF-κB pathway, while hypotaurine can rescue the inhibition of CCL20 production. Consistently, patients with high levels of ADO were usually accompanied by an overexpression of CCL20 and its only known receptor CCR6^27,28^, whereas low ADO expression was associated with low levels of CCL20 and CCR6 in gliomas (Fig. 5b; Supplementary Fig. S7; Supplementary Table 3). The original RNA-seq results suggested that glioma patients have higher levels of CCL20, CXCL1, CXCL2, CXCL5, and CXCL16 compared to noncancerous patients and that the levels of these small chemotactic cytokines in glioma are correlated with malignancy. To test this hypothesis, we performed Luminex assays (R&D Systems) to detect the concentrations of these cytokines in cerebrospinal fluid (CSF) collected from glioma patients (Fig. 5c) and nontumor patients who were suffering from hydrocephalus or intracranial hypertension but not subjected to intracranial infection or inflammation (Supplementary Table 3). Interestingly, only CCL20 displayed a significant difference (significant; *p = 0.01) in levels between glioma and nontumor patients (Fig. 5d). To investigate this further, glioma patients were divided into two groups according to their ADO levels (Fig. 5c, d). The results showed that there were trends linking these cytokines, CCL20, CXCL1, CXCL2, CXCL5, and CXCL16, to ADO expression in gliomas but these correlations were not significant, probably due to the low number of patients recruited to the study (Fig. 5d; Supplementary Table 3). Taken together, these findings indicated that ADO induces the NF-κB pathway to enhance CCL20 production to drive glioma tumorigenesis and progression.

### Inhibiting ADO attenuates glioblastoma growth in vivo

**Fig. 6:**
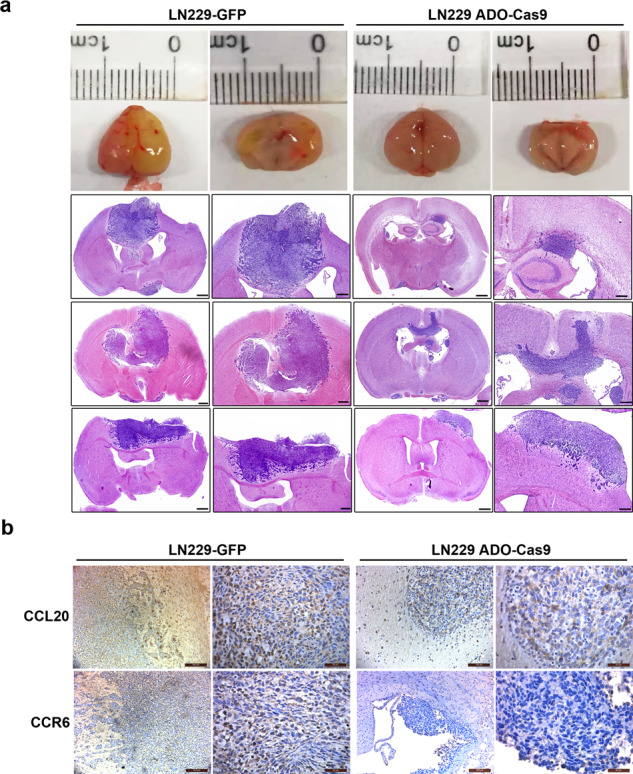
Inhibiting ADO attenuates glioblastoma growth in vivo. **a** LN229 ADO-Cas9 and control cells were injected into an orthotopical mouse xenograft model to analyze for in vivo tumor growth. Scale bars = 100 μm and 500 μm. **b** CCL20 and CCR6 expression were evaluated by immunohistochemistry in the xenograft tumors. Scale bars = 50 μm and 200 μm. Data represent three independent experiments with similar results.

We previously showed that the ADO deletion in the LN229 ADO-Cas9 cells decreases glioblastoma proliferation and self-renewal capacity in vitro (Figs. 2b and 3a). Next, we sought to determine whether the ADO axis contributes towards glioblastoma growth in vivo. To achieve this, we evaluated the effects of ADO deletion on in vivo tumor growth using an orthotopical mouse xenograft model. As demonstrated in Fig. 6, tumors derived from the LN229 ADO-Cas9 cells had a significantly decreased growth rate when compared with the control group (Fig. 6a). Consistent with in vitro and Luminex assays results, tumor tissues derived from LN229 ADO-Cas9 cells displayed conspicuously lower levels of CCL20 and CCR6 compared to the control group (Fig. 6b). Collectively, the results suggest that the ADO-hypotaurine axis targets the NF-κB pathway to promote CCL20 expression and activity in glioma tumorigenesis and progression.

## Discussion

A renewed interest in the study of glioma metabolism has led to several seminal discoveries leading to the identification of critical biomarkers for disease aggressiveness. Using a capillary electrophoresis-mass spectrometry, we have previously profiled 50 clinical samples and identified more than 247 metabolites related to glioma development. We have also found that hypotaurine as one of the top metabolites that can be used to differentiate GBM from low-grade glioma and that its intertumoral levels are associated with the expression of its biosynthetic enzyme ADO. Hitherto, few reports have examined the oncogenic role of ADO/hypotaurine in glioma. More recently, ADO has been reported to transduce the oxygen-regulated stability of proteins by the N-degron pathway to mediate cellular responses to hypoxia^25^. In the present study, we confirmed the tumorigenic role of ADO in glioma and that its expression is inversely correlated with patient prognosis. In addition, we provide the first evidence that ADO promotes the intracranial secretion of CCL20 via activating NF-κB pathway. In a reciprocal manner, accumulation of CCL20 can further stimulate NF-κB signaling^29,30^ thus forming a feedforward loop to promote cancer stemness and drive tumor progression.

CDO1 is an enzyme that catalyzes the conversion of cysteine to cysteine sulfinic acid (CSA), which is subsequently decarboxylated to hypotaurine by cysteine sulfinic acid decarboxylase (CSAD). Previous studies have demonstrated that the CDO1/CSA axis is necessary for the growth of aggressive high-grade gliomas^20,21^. ADO was initially identified as a thiol dioxygenase playing a role in cysteine metabolism, which is responsible for the synthesis of antioxidant glutathione. It has also been shown to contribute to the production of hypotaurine from cysteine in a parallel pathway to CDO1^18,19^. In here, we obtained evidence that ADO may function in parallel with the CDO1/CSA axis to drive hypotaurine production and thereby the growth of aggressive high-grade gliomas. Of particular interest, we also found that ADO regulates CDO1 expression in glioma cell lines. Furthermore, we uncovered that the expression of both the active phosphorylated p65 and total p65 NF-κB subunit is regulated by ADO and that the endogenous CCL20 levels are regulated by ADO through the NF-κB pathway. We also found that the CSF concentrations of CCL20 in glioma patients were significantly higher compared to nontumor patients. However, whether the concentration of CCL20 is associated with the degree of malignancy requires further investigation with more patient materials. This cross-talk between NF-κB and CCL20 drives tumor progression and promotes cancer stemness in multiple malignancies including glioma.

GBM is composed of heterogeneous tumor cell populations involving those with stem cell properties which contributes to therapeutic resistance and cancer initiation^31–33^. To date, numerous reports have demonstrated that NF-κB can promote GSCs via driving mesenchymal (MES) *trans*-differentiation^34^, tightly correlated with Notch1 in glioma^35^, regulating differentiation of GSCs which keeps differentiating GSCs in a proliferative astrocytic precursor state, and so on^36^. In this report, we also uncovered that ADO activates NF-κB signaling through hypotaurine to augment the secretion of a superfamily of small chemotactic cytokines, including CCL20. Accumulating evidence demonstrates that the NF-κB-CCL20 loop promotes the expansion and self-renewal of glioma-stem-like cells^29,30^. Consistent with this, we demonstrated that ADO promotes glioma stem cell phenotypes through activating the NF-κB-CCL20 axis. Chemokines secreted by the tumor, the adjacent stroma and inflammatory cells, play key roles in the recruitment of leukocytes, macrophages and immature dendritic cells to the tumor environment and are often expressed in response to NF-κB activation. Moreover, many of these chemokines can further potentiate inflammatory reactions by activating NF-κB. Thus, NF-κB not only acts as an upstream regulator of chemokines but also a downstream target and effector of cytokines^29,37,38^. Further in agreement with this notion, accumulation of CCL20 has been demonstrated to be able to promote the self-renewal and maintenance of breast cancer stem cells (BCSCs) through p38 mitogen-activated protein kinase (MAPK)-mediated activation of p65 nuclear NF-κB pathway^39^. Importantly, it has also been shown the NF-κB activation by CCL20 can in turn increase further CCL20 expression to form a positive feedback loop between NF-κB and CCL20 pathways in these breast cancer cells^39^. In concordance, the CCL20 secreted by colorectal cancer (CRC) cells can also recruit regulatory T cells (Tregs) to promote chemoresistance via a FOXO1/CEBPB/NF-κB/CCL20 signaling loop in these cells^40^. In consequence, it is likely that the high levels of CCL20 induced by ADO through the hypotaurine/NF-κB signaling axis in glioma may also function with NF-κB in a positive feedforward loop to promote cancer stemness and drive tumor progression.

In conclusion, our results demonstrate for the first time that ADO functions in a tumor initiation and progression role in glioma. In addition, ADO triggers a glioma stem cell phenotype via activating a NF-κB-CCL20 signalling axis. Collectively, the ADO/hypotaurine axis can serve as a promising target for developing effective therapeutic strategies against glioma progression.

## Materials and methods

### Cell lines and plasmids

LN229 and U118 cells were purchased from the American Type Culture Collection (ATCC), cultured according to the ATCC culture guidelines, which were maintained in Dulbecco’s modified Eagle’s medium (HyClone, Logan, UT, USA) supplemented with 5% or 10% fetal bovine serum (Gibco, Carlsbad, CA, USA) and 1% penicillin/streptomycin (Gibco) at 37˚C with 5% CO_2_.

Human ADO Gene Lentiviral ORF cDNA expression plasmid, C-GFP Spark tag ADO overexpression (OE) and Vehicle (PLV) were purchased from Sino Biological lnc. Beijing, siRNA ADO 870 and 954 were generated by GenePharma (Suzhou, China). Two siRNA sequences were designed for ADO silencing: forward 5’-GGGACAACCUGCACCAGAUTT-3’, reverse 5’-AUCUGGUGCAGGUUGUCCCTT-3’, and forward 5’-GCAUGCACGGCAUGCUCAATT-3’, reverse 5’-UUGAGCAUGCCGUGCAUGCTT-3’. ADO primer sequence: forward 5’-CCGCCAGTCACCTACATGC-3’, reverse 5’-CGTCTAGCTTGTCCATGCAG-3’. CCL20 primer sequence: forward 5’-TGCTGTACCAAGAGTTTGCTC, reverse 5’-CGCACACAGACAACTTTTTCTTT. The sgRNAs of CRISPR-Cas9-based ADO knockout: sgADO1: TTCGCGGGCCGAGTACACCG, sgADO2: CGATGTTCAAGTCCTCGGCG, targeting ADO were designed online https://zlab.bio/guide-design-resources. The two sgRNAs were then synthesized into dual-sgRNA system fragment.

### Immunohistochemistry and histopathology

This study was approved by the Medical Committee of Ethical Experiments at Affiliated Zhongshan Hospital of Dalian University. Human tissue samples were collected from Affiliated Zhongshan Hospital of Dalian University with informal patient consent (Supplementary Table 1). The expression levels of ADO was scored semiquantitatively based on staining intensity and distribution using the immunoreactive score (IRS). Briefly, IRS = SI (staining intensity) × PP (percentage of positive cells). SI was assigned as: 0 = no brown particle staining, 1 = light brown particles, 2 = moderate brown particles, and 3 = dark brown particles. PP was defined as: 0 for <10% positive cells, 1 = 10–40% positive cells, 2 = 40–70% positive cells, and 3 for ≥70% positive cells. The two scores were multiplied and used to determine high (score ≥ 3) or low (score ≤ 3) expression of ADO.

The tissue microarrays (TMA) containing 180 cases of glioma tissues were purchased from Outdo Biotech Company (Shanghai, China) and stained with ADO (1:500) antibody. Immunostaining and analysis were all performed by Outdo Biotech Company. The patient information was listed in Supplementary Table 2.

### Spheroid formation assay

LN229 cells were plated in ultra-low adhesion 96-well culture plates with serum-deprived DMEM/F12, which contained 20 ng/ml basic FGF, 20 ng/ml EGF, and a proportion of B27 in medium (1:50 v/v) for 10 days.

### Immunoblotting

LN229, U118 cells or glioma tissues were collected for protein extraction and detection of ADO, CDO, Sox2, Oct4, GAPDH, phosphorylated p65, and total p65 using a protein extraction kit (KeyGEN Biotechnology, Nanjing, China). Proteins (20–30 μg per lane) were loaded on a 8–12% SDS-polyacrylamide electrophoresis gel to separate, then transferred onto the nitrocellulose membrane. The membrane was blocked using 5% fat-free milk dissolved in Tris-buffered saline with Tween-20 (TBS-T) for 2 h at room temperature and subsequently incubated overnight with primary antibodies against ADO (Proteintech Group, 16479-1-AP, China and Santa Cruz Inc, sc-515318, USA), CDO1 (Proteintech Group, 12589-1-AP, China), Sox2 (abcam, ab92494 and Cell Signaling, 3579 S), Oct4 (abcam, ab109183 and Cell Signaling, 2890 S), phosphorylated p65 (Cell Signaling, 3033 S), total p65 (Cell Signaling, 8242 S), CCL20 (abcam, ab9829), CCR6 (abcam, ab227036) and then incubated the corresponding secondary antibodies.

### Immunofluorescence assay

At indicated differentiation times, LN229 cells were washed with phosphate buffered saline (PBS) twice and fixed with 4% para-formaldehyde/PBS for 10 min at room temperature, subsequently permeabilized in 0.5% Triton X-100 and incubated in 2% Bovine Serum Albumin (BSA). Afterward, cells were nurtured with the primary antibodies overnight at 4 °C and then incubated with the secondary antibodies for 1 h at room temperature. DAPI was used to stain the nucleus.

### Luminex and Elisa assay

Luminex analysis for a superfamily of small chemotactic cytokines containing CCL20, CXCL1, CXCL2, CXCL5, and CXCL16 was purchased from Univ Biotech Company (Shanghai, China), the detail report was displayed in Supplementary Table 3. Elisa assay was performed as instruction of Human CCL20/MIP-3 alpha ELISA Kit (Proteintech Group, KE00149, China) as described.

### Migration assay

Migration assays were performed using Corning Boyden chambers (24-well insert, 8 μm, Corning, NY, USA). Approximately 20,000 cells were seeded with serum-free DMEM in the Transwell Permeable Support, 8.0 μm polycarbonate membrane, 6.5 mm inserts (Pittsburg Corning, UK). The bottom part of the well was filled with DMEM with 10% FBS in the lower chamber as the chemoattractant. 24 h after seeding, cells migrated although the pores to the bottom surface of the transwell were fixed with 4% para-formaldehyde, washed with PBS and stained with 0.5% crystal violet for visualization.

### Animal experiments

All the experiment were performed with the approval and survey of The Animal Ethics Committee of Affiliated Zhongshan Hospital of Dalian University. Four to six weeks BALB/c nude mice were feeding in strict accordance with the rules of the SPF Experimental Animal Center of Affiliated Zhongshan Hospital of Dalian University. 1 × 10^5^ LN229-GFP or LN229 ADO-Cas9 cells were resuspended and stereotactically implanted into frontal lobe of the mice. Animals were monitored and sacrificed when neurological signs appeared.

### Statistical analysis

The mean ± standard deviation (SD) was used to articulate the results. Date assorted and estimated in normality with the comparison of two groups via Student’s *t*-test. Treatments and control groups were assessed and analyzed with the aid of one-way ANOVA. Graph Pad Prism software was used for the execution of the statistic assays. *P* ≤ 0.05 was supposed as an indication of statistical significance.

## Supplementary information

Supplementary Figure S1

Supplementary Figure S2

Supplementary Figure S3

Supplementary Figure S4

Supplementary Figure S5

Supplementary Figure S6

Supplementary Figure S7

Supplementary Figure Legends

Supplementary Table 1

Supplementary Table 2

Supplementary Table 3
