# Supplementary figures and images for: ADO/hypotaurine: a novel metabolic pathway contributing to glioblastoma development
Source: Cell Death Discov. 2021 Jan 22;7:21. doi: 10.1038/s41420-020-00398-5 (PMC7822925; doi:10.1038/s41420-020-00398-5)

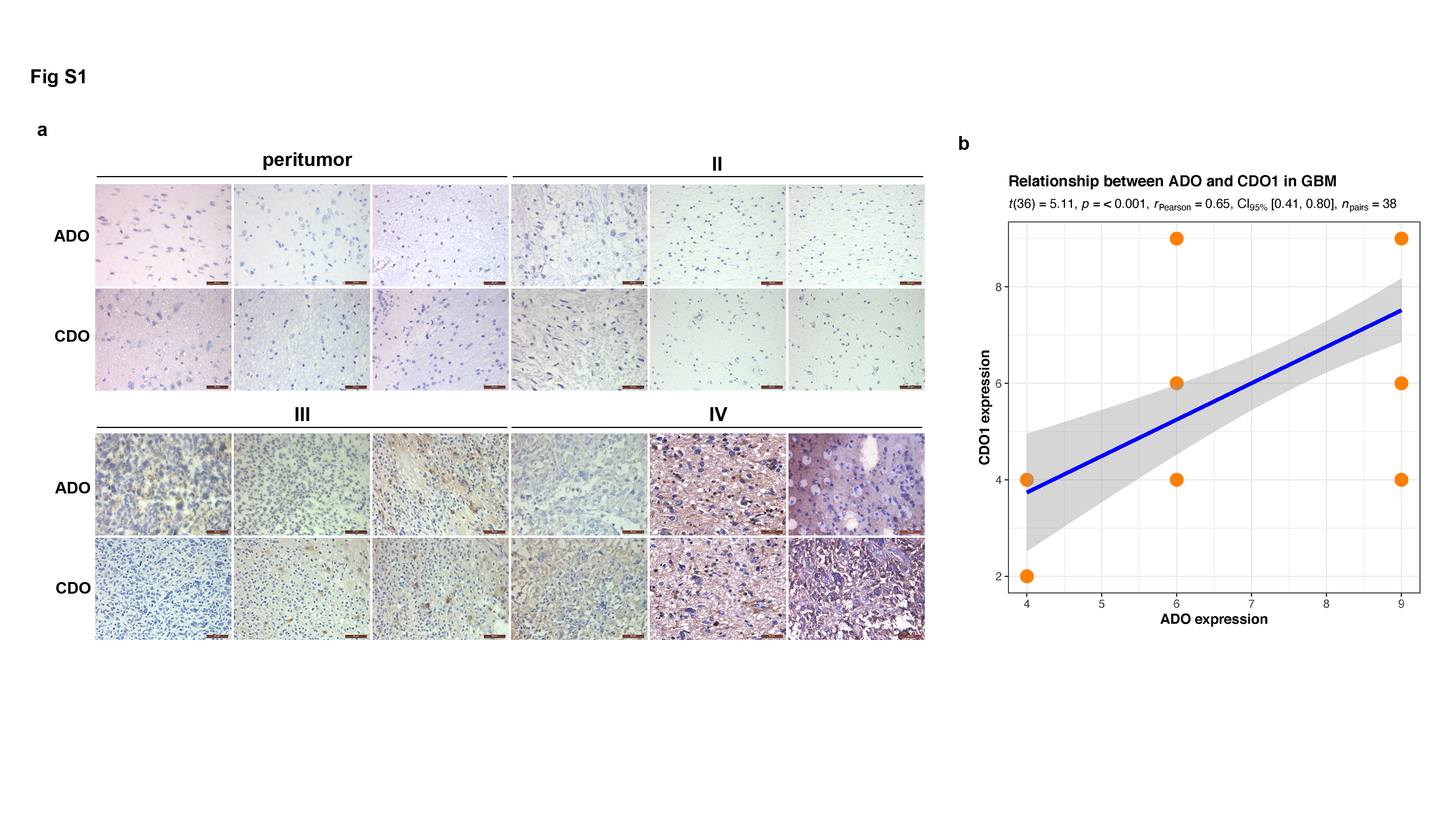

Supplement: Supplementary file 1 — Supplementary Figure S1 [file 41420_2020_398_MOESM1_ESM.tif]

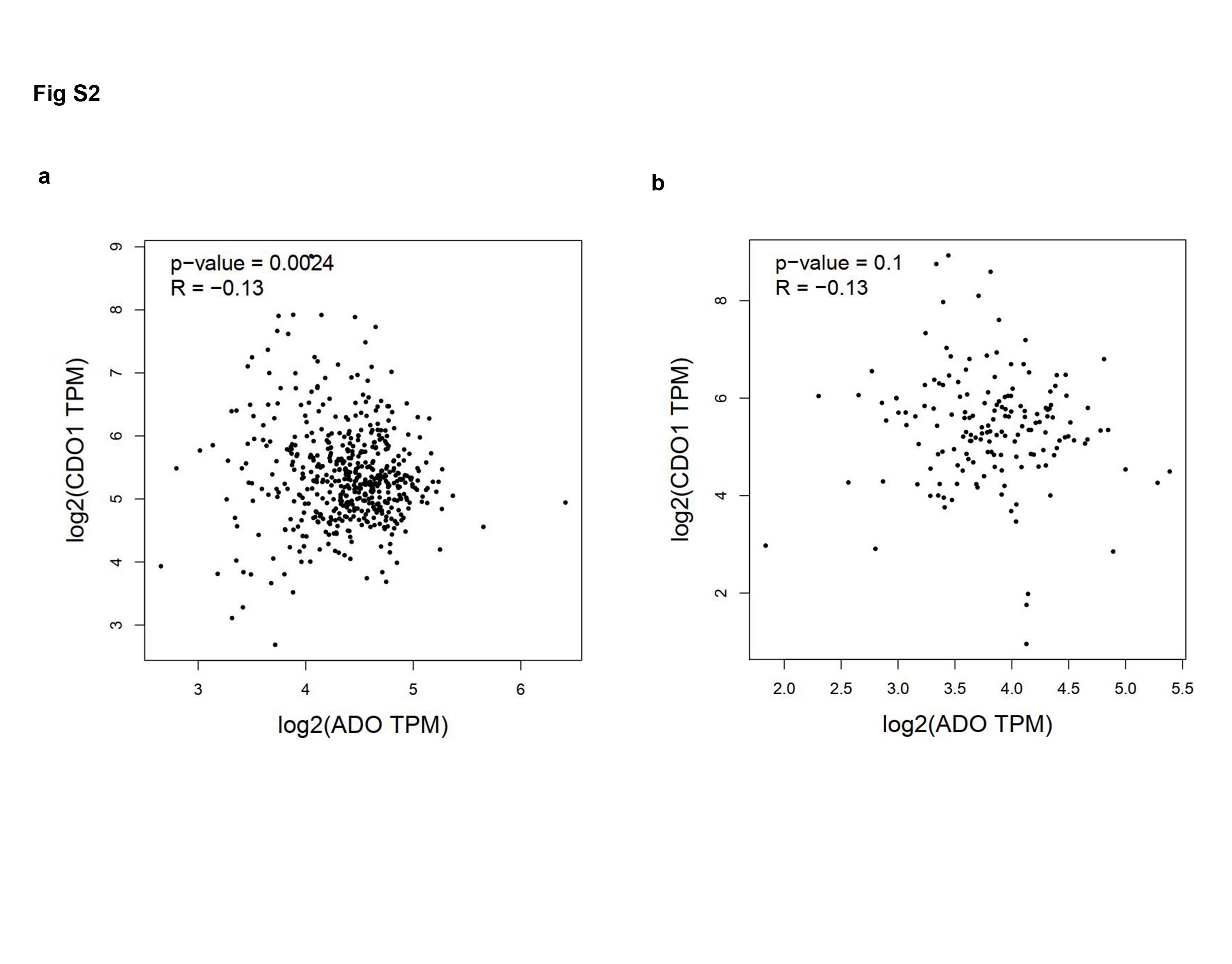

Supplement: Supplementary file 2 — Supplementary Figure S2 [file 41420_2020_398_MOESM2_ESM.tif]

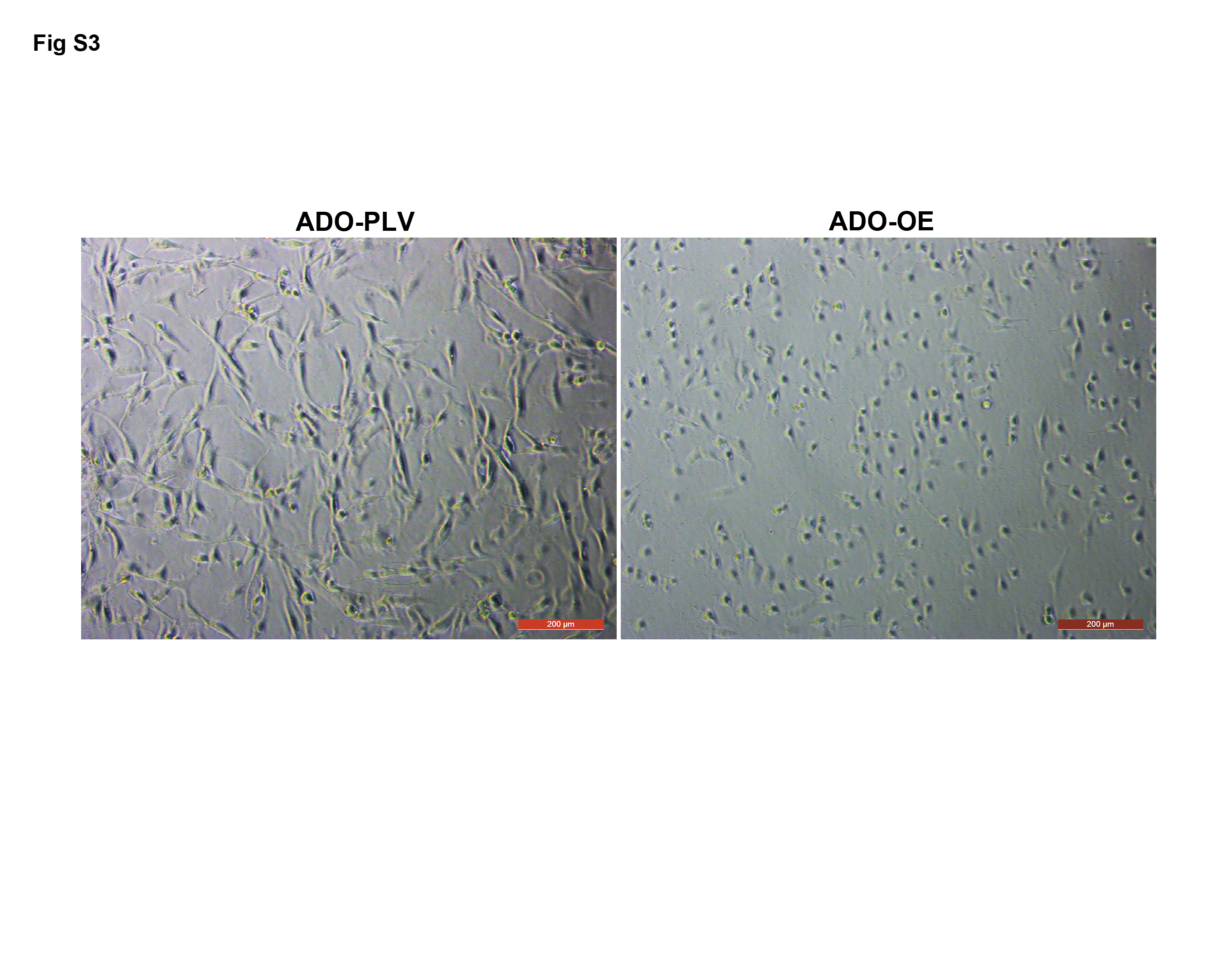

Supplement: Supplementary file 3 — Supplementary Figure S3 [file 41420_2020_398_MOESM3_ESM.tif]

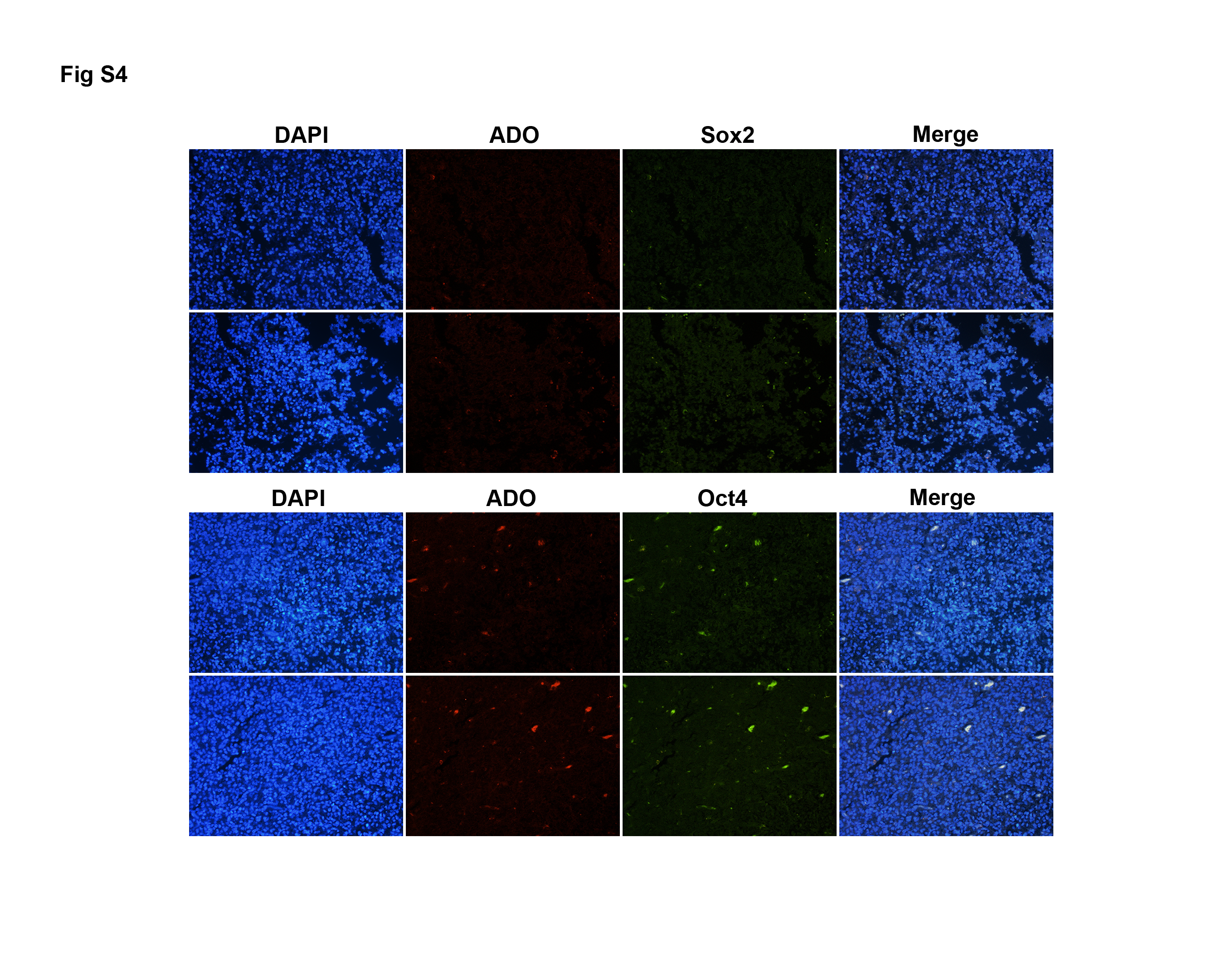

Supplement: Supplementary file 4 — Supplementary Figure S4 [file 41420_2020_398_MOESM4_ESM.tif]

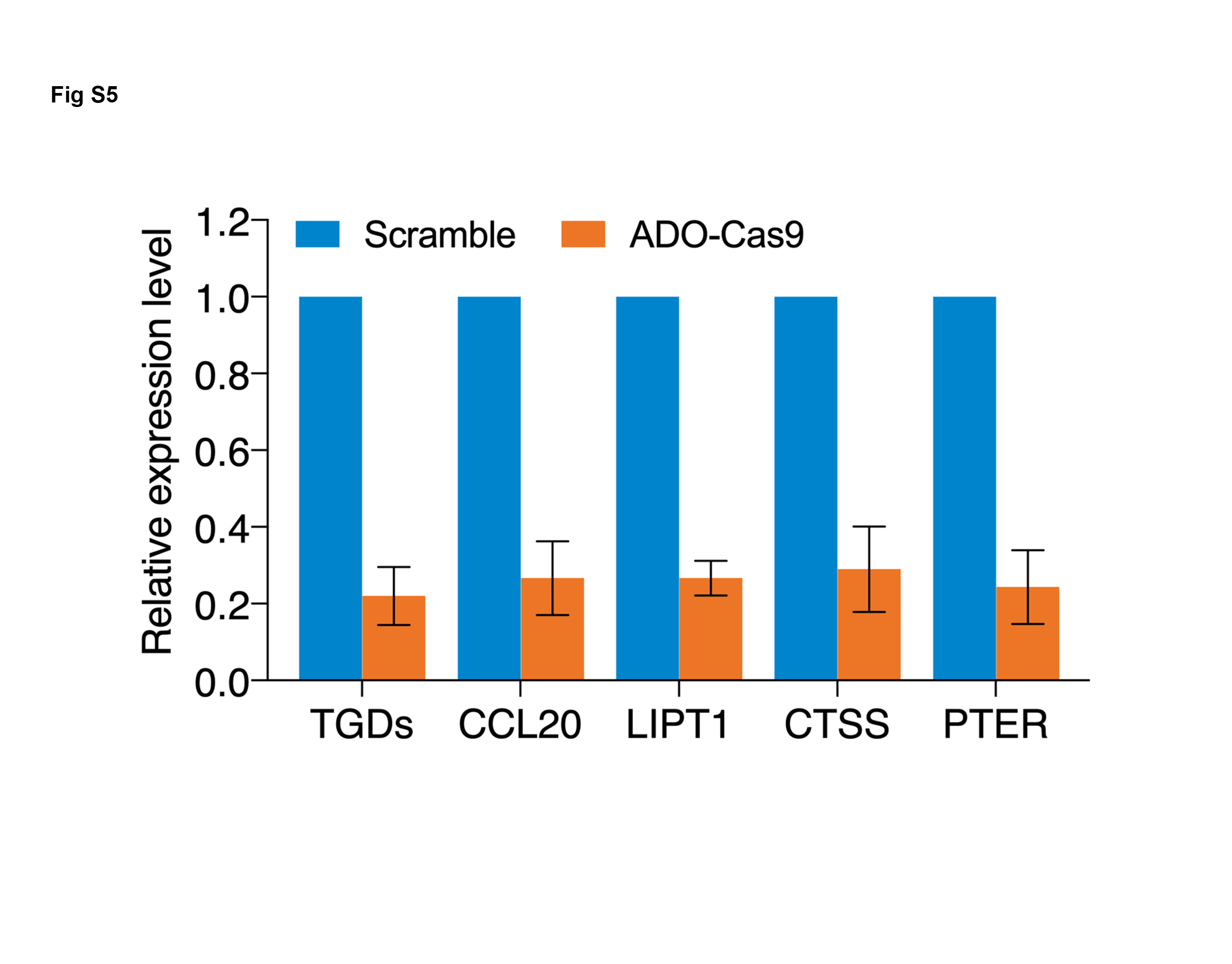

Supplement: Supplementary file 5 — Supplementary Figure S5 [file 41420_2020_398_MOESM5_ESM.tif]

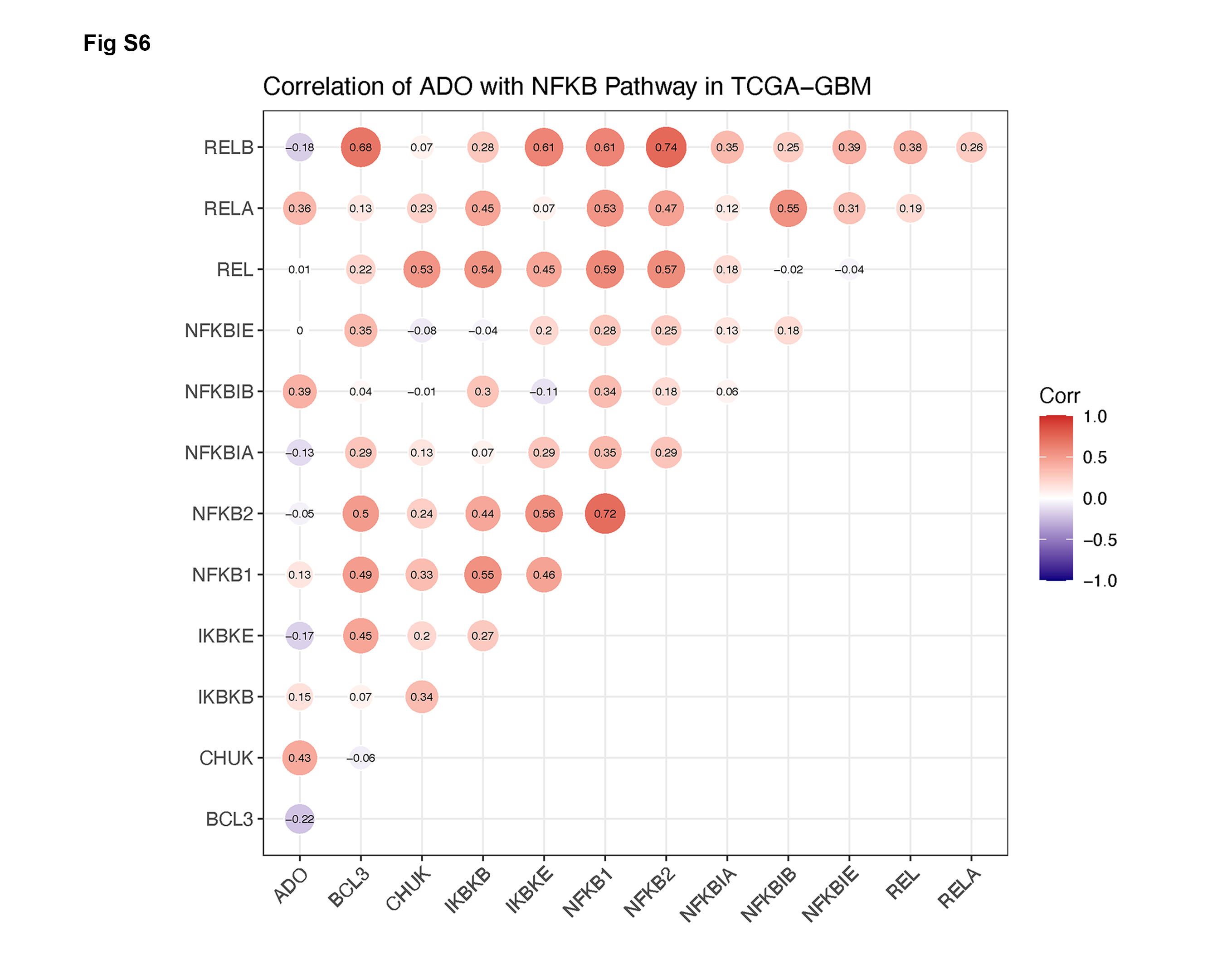

Supplement: Supplementary file 6 — Supplementary Figure S6 [file 41420_2020_398_MOESM6_ESM.tif]

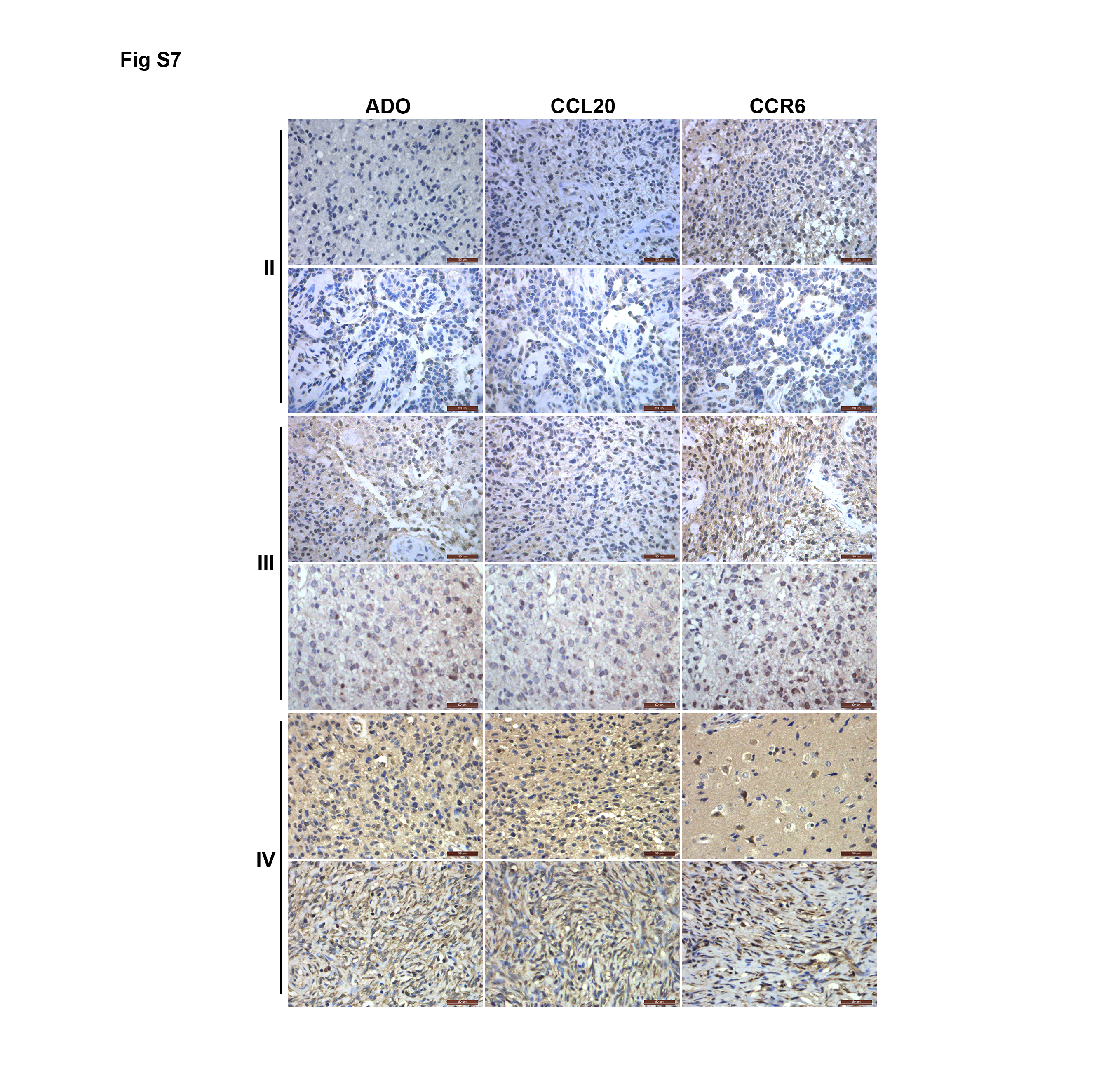

Supplement: Supplementary file 7 — Supplementary Figure S7 [file 41420_2020_398_MOESM7_ESM.tif]
