# Supplementary material for: ADO/hypotaurine: a novel metabolic pathway contributing to glioblastoma development
Source: Cell Death Discov. 2021 Jan 22;7:21. doi: 10.1038/s41420-020-00398-5 (PMC7822925; doi:10.1038/s41420-020-00398-5)
Supplement: Supplementary file 8 — Supplementary Figure Legends [file 41420_2020_398_MOESM8_ESM.docx]

Supplementary legends

Fig S1. An association between ADO and CDO1 expression levels in glioma

patients with different tumor grades. a, Representative ADO and CDO1 staining images from 38 pairs of peritumor and tumor patient samples. b, Correlation between ADO and CDO1 expression in 38 pairs of peritumor and tumor patient samples.

Fig S2. a, Weak negative correlations between ADO and CDO1 mRNA levels in low grade glioma LGG (518 cases) (R=-0.13; **p=0.0024) and b, glioblastoma GBM (163cases ) (R=-0.13; p=0.1) by GEPIA using TCGA datasets.

Fig S3. Morphological changes in U118 cells upon ADO overexpression. ADO overexpression in U118 cells caused long spindly cells to change to more rounded cells.

Fig S4. Patients with low levels of ADO expression were accompanied by low levels of Sox2 and Oct4 expression. Representative immunofluorescent Sox2 (upper panel) and Oct4 (lower panel) staining images are shown.

Fig S5. RNA expression levels of the top five significant candidates from RNA-seq analysis in LN229 ADO-Cas9 cells versus control LN229 cells.

Fig S6. A strong correlation between the expression levels of ADO and NF-κB pathway members in the TCGA-GBM database.

Fig S7. Serial sections from patients were used to study ADO, CCL20 and CCR6 expression by immunohistochemistry. Scale bars=50 μm. Representative staining patterns are shown.
